# Supplementary material for: Structure and inference in hypergraphs with node attributes
Source: Nat Commun. 2024 Aug 16;15:7073. doi: 10.1038/s41467-024-51388-5 (PMC11329712; doi:10.1038/s41467-024-51388-5)
Supplement: Supplementary file 1 — Supplementary Information [file 41467_2024_51388_MOESM1_ESM.pdf]

Supplementary Materials for
Structure and inference in hypergraphs with node attributes

Anna Badalyan, Nicolò Ruggeri, Caterina De Bacco

A. SYNTHETIC DATA GENERATION

We generated synthetic networks using the sampling algorithm of Hy-MMSBM (1), with implementation as in the `HGX` library described in (2). We set parameters as follows: $N = 500$, $|E| = 2720$ and $K = \{2, 3, 5, 10\}$. We specify the number of hyperedges of each size using the dimension sequence `dim_seq = {2: 300, 3: 300, 4: 200, 5: 200, 6: 150, 7: 150, 8: 150, 9: 150, 10: 120, 11: 120, 12: 120, 13: 120, 14: 100, 15: 100, 16: 100, 17: 100, 18: 80, 19: 80, 20: 80}`.

The attributes were generated to match the community structure. In all experiments, we set $Z = K$, and produce the attribute matrix X as follows. First, the matrix X is initialized equal to the community assignments u of the nodes. Then, for a fraction ρ of the nodes, we replace the corresponding attribute with a random one. We perform experiments with ρ ranging in $\{0.1, 0.2, 0.3, 0.4, 0.5, 0.6, 0.7, 0.8, 0.9\}$.

We randomly generated 10 instances of higher-order networks and 10 instances of attributes for each configuration. The value of γ used in these experiment was equal to the proportion of non-shuffled attributes $\gamma = 1 - \rho$.

B. SOLVING FOR THE MEMBERSHIP MATRIX UPDATES

We have the following equation to solve in order to find u_{ik}:

$$a_{ik}\, u_{ik}^2 - (a_{ik} + b_{ik} + c_{ik})\, u_{ik} + b_{ik} = 0 \quad , \tag{S1}$$

where a_{ik}, b_{ik}, c_{ik} are all positive values.
This expression can be presented as a general quadratic equation

$$ax^2 - bx + c = 0 \quad , \tag{S2}$$

where a, b, c are positive numbers and $b > a + c$. The resulting discriminant Δ is given by

$$\Delta = b^2 - 4ac > (a + c)^2 - 4ac = (a - c)^2 > 0 \quad . \tag{S3}$$

Hence the discriminant is positive and there exist two distinct and real solutions to the equation.

Now we show that the smallest root $x_0 = \frac{b-\sqrt{\Delta}}{2a}$ satisfies the constraints on u, that is $0 \leq x_0 \leq 1$. The fact that $x_0 \geq 0$ derives directly from the fact that $b \geq \sqrt{\Delta}$.
Then, we show that $x_0 < 1$:

$$\begin{aligned} &\frac{b - \sqrt{\Delta}}{2a} < 1 \\ \iff\ & b - \sqrt{\Delta} < 2a \\ \iff\ & b^2 - 4ab + 4a^2 < \Delta \\ \iff\ & a + c < b \quad . \end{aligned}$$

Similarly, it can be shown that the root $x_1 = \frac{b+\sqrt{\Delta}}{2a}$ does not yield a valid update for u, as

$$\frac{b + \sqrt{\Delta}}{2a} < 1 \iff a + c > b \, ,$$

which is never satisfied.

C. ALTERNATIVE FORMULATION FOR EXCLUDING ATTRIBUTES

In the main manuscript we have described a model that allows a node to have multiple values for one attribute type. While in certain cases attributes could be excluding, e.g. age can take only one value, this can still be handled by that model. Alternatively, one can modify the model by assuming a probability distribution that explicitly imposes the choice of only one value, e.g. with a Multinomial distribution. Here we illustrate how our model can be adapted to this case and highlight the main differences with the formulation adopted in the main manuscript.

We assume that each entry X_{iz} is extracted from a Multinomial distribution with parameter $\pi_{iz} = \sum_{k=1}^{K} u_{ik}\, \beta_{kz}$. Then, the likelihood of the attributes matrix can be modelled as:

$$P_X(X|U,\beta) = \prod_{i \in V} Mult\,(X_i;\pi_i) \quad , \tag{S4}$$

which gives the following log-likelihood for the attributes:

$$L_X(U,\beta) = \sum_{i=1}^{N}\sum_{z=1}^{Z} x_{iz}\log(\pi_{iz}) = \sum_{i=1}^{N}\sum_{z=1}^{Z} x_{iz}\log\left(\sum_{k=1}^{K}\beta_{kz}u_{ik}\right) . \tag{S5}$$

Using a standard variational approach to lower bound the log-likelihood we get:

$$\mathcal{L}_X(U,\beta,h) = \sum_{i,z,k} x_{iz}\left[h_{izk}\log(\beta_{kz}u_{ik}) - h_{izk}\log(h_{izk})\right] \tag{S6}$$

with the equality reached when

$$h_{izk} = \frac{\beta_{kz}u_{ik}}{\sum_{k'}\beta_{k'z}u_{k'z}} . \tag{S7}$$

While Eq. (S6) looks simpler than the one we derived using the Bernoulli distribution, the main challenge is to obtain a tractable solution for the updates of u_{ik} as we introduce constraints on the parameters. The constraint for β is $\sum_z \beta_{kz} = 1,\ \forall k$. However, now the constraint on u_i involves all of the entries of this vector at the same time: $\sum_k u_{ik} = 1$, increasing the complexity of the subsequent derivations. In addition, we still impose positivity $u_{ik} \geq 0$, $\forall i,k$.
By introducing Lagrange multipliers $\lambda = (\lambda^{(\beta)}, \lambda^{(u)}, \mu^{(u)})$, where $\lambda^{(u)}$ controls the summation to one term and $\mu^{(u)}$ positivity, we get the following update for u_{ik}:

$$u_{ik} = \frac{(1-\gamma)\sum_{e\in E: i\in e} A_e \sum_{j\neq i \in e}\sum_q \rho_{ijkq}^{(e)} + \gamma\sum_z x_{iz}h_{izk}}{\lambda_i^{(u)} - \mu_{ik}^{(u)} + (1-\gamma)C\sum_{j\in V, j\neq i}\sum_{q=1}^{K} u_{jq}w_{kq}} \quad . \tag{S8}$$

To estimate $\lambda^{(u)}$ we need to solve the following equation:

$$\sum_{k=1}^{K} u_{ik} = \sum_{k=1}^{K}\frac{(1-\gamma)\sum_{e\in E: i\in e} A_e \sum_{j\neq i \in e}\sum_q \rho_{ijkq}^{(e)} + \gamma\sum_z x_{iz}h_{izk}}{\lambda_i^{(u)} - \mu_{ik}^{(u)} + (1-\gamma)C\sum_{j\in V, j\neq i}\sum_{q=1}^{K} u_{jq}w_{kq}} = 1 \quad . \tag{S9}$$

Equation (S9) cannot be solved in closed-form but can be solved numerically, e.g. with root-finding methods. However, this can slow down the implementation considerably and may not always converge to a solution.

D. THE ADVANTAGES OF USING A HYPERGRAPH REPRESENTATION

To demonstrate possible advantages of utilizing a hypergraph representation, and specifically of enriching it with node attributes, we compare the performance of HyCoSBM against those of a dyadic representation of a hypergraph on a hyperedge prediction task.

There are various ways that one can use to project an hypergraph into a standard network structure with pairwise edges. Here we consider the clique expansion, where for each hyperedge one creates a clique with all the possible pairs of nodes in it. The resulting network is the union of these cliques and edge weights are the numbers of hyperedges in

which a pair of nodes was contained in. This is a popular approach when investigating hypergraphs, see for example (3, 4). We then apply an algorithm that has similar characteristics as HyCoSBM but is only valid in networks. As the focus of this work is on utilyzing node attributes as additional information, as a comparison we use MTCOV (5), a probabilistic model for networks that is able to utilize node attributes to efficiently infer the network structure. Similarly to HyCoSBM, it also uses latent variables like community memberships. As approaches for (pairwise) networks only output the probability of observing pairwise interactions, we define the probability of a hyperedge as the product of all edges belonging to its clique expansion. We refer to this approach of using MTCOV on the clique expansion as Clique-Exp.

Dataset	N	$\|E\|$	$\|E_2\|$	$\|E_{\text{Clique-Exp}}\|$
High School	327	7818	5498	5818
Hospital	75	1825	1108	1139
Primary School	242	12704	7748	8317
Workplace	92	788	742	755
Gene Disease	9262	3128	886	2837026
NYC taxi trips Mon-Tue 17-19	214	523	64	18568
NYC taxi trips Sat-Sun 00-02	214	476	53	16146

Supplementary Table I: **Statistics on graphs obtained by clique expansion**. Number of nodes N, number of hyperedges $|E|$, number of hyperedges $|E_2|$ of size 2, and number of (dyadic) edges $|E_{\text{Clique-Exp}}|$ obtained by clique expansion are reported. The latter three quantities consider the number of unique edges, not accounting for edge weights.

As a preliminary analysis, in Supplementary Table I we compare the number of interactions observed in different real-world datasets and their relative clique expansion. In contacts datasets, we observe that the majority of interactions are pairwise, with the bulk of the interactions being of sizes two and three. In addition, many higher-order edges overlap, as they contain pairs of nodes that are already present in other hyperedges. As a result, the number of unique hyperedges $|E|$ is larger than the number of unique pairwise edges in the clique expansion $|E_{\text{Clique-Exp}}|$.

This could be a reason for not observing a significant difference between Clique-Exp and HyCoSBM on the High School and Primary School datasets in predicting hyperedges. Nevertheless, it is difficult to draw a general conclusion as there are several variables that could contribute to prediction performance (e.g. how hyperedges overlap by sharing subset of nodes, etc...). For instance, in other datasets similar to the contacts in schools, HyCoSBM outperforms Clique-Exp; this happens in Hospital with AUC equal to 0.776 versus 0.714 and in Workplace with respective AUC scores of 0.81 and 0.774, as reported in Supplementary Table II.

Dataset	Attribute	Z	HyCoSBM			Hy-MMSBM		Clique-Exp		
			K	γ	AUC	K	AUC	K	γ	AUC
Gene Disease	DPI	25	30	0.500	0.9 ± 0.07	2	0.84 ± 0.122	5	0.995	0.682 ± 0.015
High School	class	9	11	0.995	0.899 ± 0.011	24	0.884 ± 0.006	24	0.995	0.906 ± 0.008
	has filled questionnaire	2	21	0.800	0.892 ± 0.013			29	0.200	0.894 ± 0.007
	has facebook	2	15	0.950	0.888 ± 0.008			30	0.800	0.892 ± 0.013
	sex	2	16	0.800	0.889 ± 0.009			25	0.600	0.895 ± 0.009
Primary School	class	11	10	0.600	0.841 ± 0.013	11	0.841 ± 0.007	24	0.995	0.847 ± 0.010
	sex	2	12	0.200	0.841 ± 0.007			23	0.100	0.836 ± 0.007
Hospital	status	4	2	0.200	0.776 ± 0.032	2	0.758 ± 0.016	23	0.995	0.714 ± 0.046
Workplace	department	5	5	0.995	0.81 ± 0.02	5	0.752 ± 0.039	6	0.990	0.774 ± 0.025

Supplementary Table II: **AUC scores achieved by HyCoSBM, Hy-MMSBM, Clique-Exp**. The best results achieved by 5-fold cross validation as well as best γ and K by all models are reported. For the Clique-Expon Gene Disease dataset, the maximum number of communities used during cross-validation was $K = 7$ due to computational constraints. AUC values and errors are averages and standard deviations over 5 cross-validation folds.

On the other hand, the clique expansion obtained from Gene Disease contains about 2.8 million dyadic edges, compared to only 3128 hyperedges. This is because it contains many hyperedges of large sizes (also of size ~ 1000 nodes). This makes it difficult to run a code on the clique expansion, even when the complexity is only linear in the number of edges, as it is the case for MTCOV. We were able to run the cross-validation procedure only for small

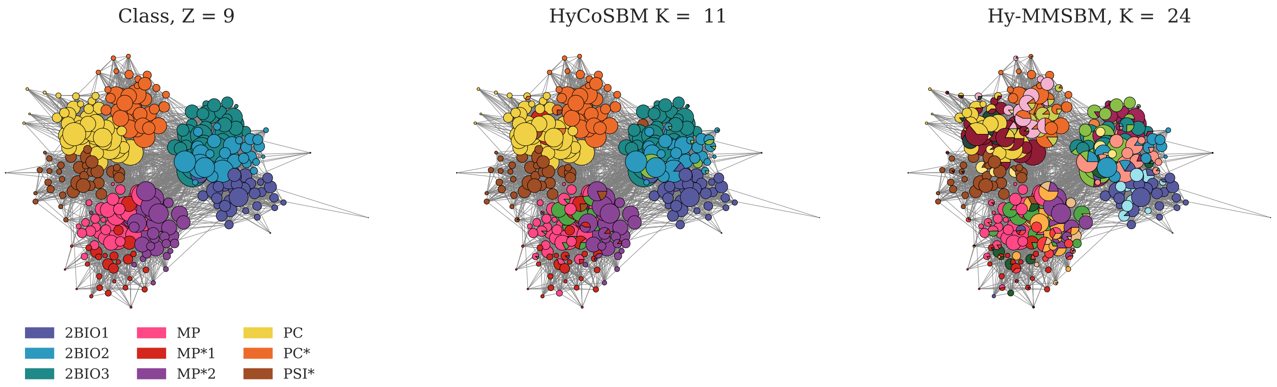

Supplementary Figure 1: Communities detected in a High School dataset of close-proximity interactions. We give the whole dataset as input to the algorithms, and compare the inferred communities against the `class` attribute (top left). The plot shows that both HyCoSBM and Hy-MMSBM detect communities aligned with the attribute, but with a number of communities greater than the number of attribute values. AUC values are slightly higher for HyCoSBM, see Table III in the main manuscript.

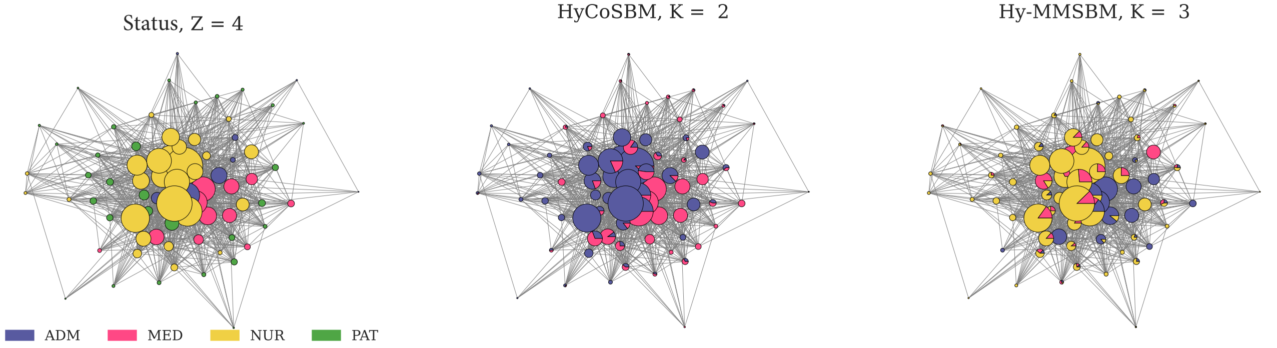

Supplementary Figure 2: Communities detected in Hospital dataset using 60% of hyperedges. We give in input to the algorithms 60% of hyperedges and compare the inferred communities against the attribute `status` (NUR=paramedical staff; PAT=Patient; MED=Medical doctor; ADM=administrative staff) (top left). This plot shows that both HyCoSBM and Hy-MMSBM detect fewer communities than the division indicated by attributes, with HyCoSBM achieving a higher AUC that Hy-MMSBM, see Fig. 2 in the main manuscript.

values of $K \leq 7$, which results in poor performance of the model, compared to both HyCoSBM and Hy-MMSBM. This shows that clique expansions could be significantly limiting, as one may not even be able to run a standard network model on them due to computational challenges. This is particularly the case in hypergraphs with large hyperedge sizes. On the contrary, both HyCoSBM and Hy-MMSBM are not significantly impacted by this and are efficient to run on large and sparse hypergraphs.

E. ADDITIONAL RESULTS OF COMMUNITY DETECTION

We provide additional results about communities detected in the High School and Hospital datasets in Supplementary Figure 1 and 2.

REFERENCES

[1] N. Ruggeri, F. Battiston, C. De Bacco, Framework to generate hypergraphs with community structure. *Phys. Rev. E* **109**, 034309 (2024).

[2] Q. F. Lotito, M. Contisciani, C. De Bacco, L. Di Gaetano, L. Gallo, A. Montresor, F. Musciotto, N. Ruggeri, F. Battiston, Hypergraphx: a library for higher-order network analysis. *Journal of Complex Networks* **11**, cnad019 (2023).
[3] P. S. Chodrow, N. Veldt, A. R. Benson, Generative hypergraph clustering: From blockmodels to modularity. *Science Advances* **7**, eabh1303 (2021).
[4] M. Contisciani, F. Battiston, C. De Bacco, Inference of hyperedges and overlapping communities in hypergraphs. *Nature communications* **13**, 7229 (2022).
[5] M. Contisciani, E. A. Power, C. De Bacco, Community detection with node attributes in multilayer networks. *Scientific reports* **10**, 15736 (2020).
